# Theoretical optimisation of a novel gas sensor using periodically closed resonators

**DOI:** 10.1038/s41598-024-52851-5

**Published:** 2024-01-30

**Authors:** Zaky A. Zaky, M. Al-Dossari, Arvind Sharma, Ahmed S. Hendy, Arafa H. Aly

**Affiliations:** 1 TH-PPM Group, Physics Department, Faculty of Science, Beni-Suef University, Beni-Suef, 62521 Egypt; 2 Department of Physics, Faculty of Science, King Khalid University, 62529 Abha, Saudi Arabia; 3 Department of Physics, Government Lohia College, Churu, Rajasthan 331001 India; 4 Department of Computational Mathematics and Computer Science, Institute of Natural Sciences and Mathematics, Ural Federal University, 19 Mira St., Yekaterinburg, 620002 Russia

**Keywords:** Computational science, Sensors and biosensors, Computational methods

## Abstract

This study investigates using the phononic crystal with periodically closed resonators as a greenhouse gas sensor. The transfer matrix and green methods are used to investigate the dispersion relation theoretically and numerically. A linear acoustic design is proposed, and the waveguides are filled with gas samples. At the center of the structure, a defect resonator is used to excite an acoustic resonant peak inside the phononic bandgap. The localized acoustic peak is shifted to higher frequencies by increasing the acoustic speed and decreasing the density of gas samples. The sensitivity, transmittance of the resonant peak, bandwidth, and figure of merit are calculated at different geometrical conditions to select the optimum dimensions. The proposed closed resonator gas sensor records a sensitivity of 4.1 Hz m^−1^ s, a figure of merit of 332 m^−1^ s, a quality factor of 113,962, and a detection limit of 0.0003 m s^−1^. As a result of its high performance and simplicity, the proposed design can significantly contribute to gas sensors and bio-sensing applications.

## Introduction

Periodic structures in optic and acoustic systems have emerged as an active field of scientific research in the last decades in different applications, such as sensors^1–5^, filters^6^, energy harvesting^7^, and smart windows^8^. Moreover, different equipment, buildings, and bridges are disturbed by different vibration sources and elastic waves in their surrounding environment. So, the fantastic mechanical and physical properties of phononic crystals (PnCs) remain a fascinating research domain due to their ability to control the wave propagation of elastic wave or vibration sources^9^. PnCs are artificial structures of unit cells with periodic Young’s modulus and mass density.

The Bragg or phononic band gap (Pn-BG), which represents the range of frequencies in which the real component of wavenumbers does not coincide in dispersion curves, is the main competitive advantage of PnC. The Pn-BGs prevent the transfer of energy through the PnCs because of the multiple destructive interferences due to the repetitive change in the acoustic impedance of the structure^10^. Furthermore, by introducing a defect layer or unit cell with different geometric, elastic properties or geometric properties into a PnC, a narrow defect mode appears within the PnBG due to the localization of energy^11^.

The acoustic resonant frequencies in fluid-filled cavities are commonly used for calculating the properties of gases or liquid substances^12^. Cylindrical or rectangular resonance devices can be constructed from glass or stainless steel as a stiff material. The shift in the resonant wavelength or frequency confined in a fluid-filled layer or cavity is highly influenced by changes in the sound speed of the fluid. In this connection, PnCs have engaged severe attention in various sensing attempts^13,14^. Recently, numerous scientific research has been done on using defective one-dimensional PnCs (1D-PnCs) with periodic materials in sensing applications^15–17^.

Taha et al.^18^ designed a 1D-PnC sensor to detect the presence of NaCl in a water sample. The structure of this model is composed of two mirror PnCs separated by a cavity. In 2022, Zaki et al.^19^ suggested a PnC sensor based on a 2D-materials. The structure consists of four periods of MoS_2_/PtSe_2_ PnCs containing a defect cavity at the center. Such structures of periodic materials need relatively high techniques and quality to be fabricated. Wang et al.^20^ demonstrated a locally resonant BG at low longitudinal elastic frequencies in harmonic oscillators connected to a slender beam in a quasi-1D configuration.

This paper investigates a novel gas sensor using periodically closed resonators. This novel study provides new information about using periodically closed resonators as greenhouse gas sensors. Furthermore, this study ensures that PnC sensors with closed resonators perform better than PnC sensors with periodic tubes^11^. A new generation of tube-adapted sensors is an urgent requirement in industry, medicine, and biology because cylindrical structures are the most common shapes in nature that are used for fluid (liquids and gas) transport. The proposed sensor can be used as a factory chimney and continuously detect the concentration of hazardous gases’ path through it. Besides, the structure can transport gases that we need to characterize simultaneously. This sensor can be manufactured more easily than nano-sensors that require high-quality and expensive techniques.

## Sensor array and modeling assumptions

**Figure 1:**
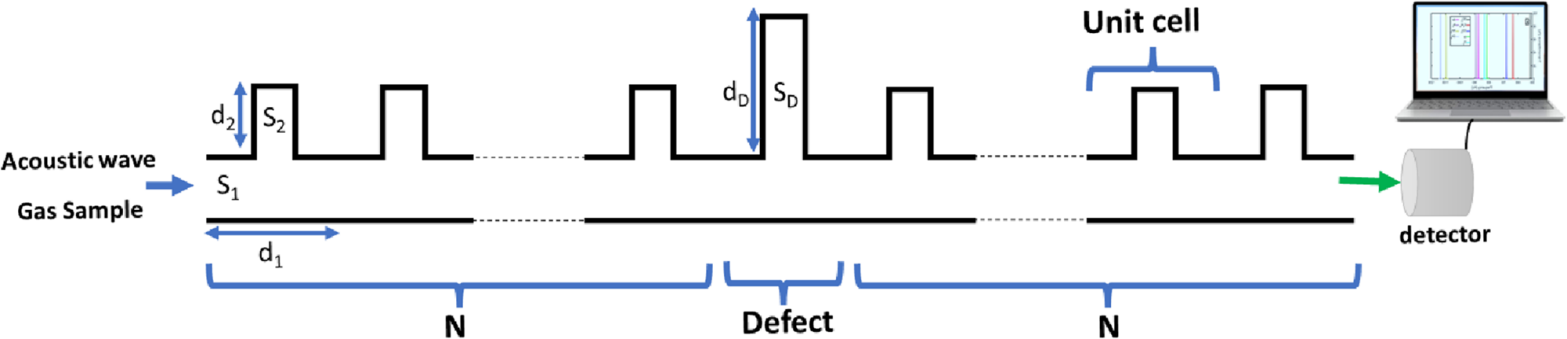
Defected 1D-PnC composed of closed resonators.

In Fig. 1, a unit cell of a closed resonator is repeated for *N* periods as a 1D-PnC. Each unit cell has four geometrical parameters, including the cross-section (*S*_*1*_) of the primary waveguide, the length (*d*_*1*_) of the primary waveguide, the cross-section (*S*_*2*_) of the finite waveguide, and the height (*d*_*2*_) of the finite waveguide. The proposed sensor is made up of two PnCs of *N* unit cells separated by a defect unit cell with the same geometry of the primary waveguide but different geometry of the finite waveguide.

### Using the transfer matrix method

The incident wave may be deemed a plane wave for sufficiently large wavelengths. The unimodular acoustic transfer matrix method (UATMM) is used to investigate the interaction between acoustic waves and structures^21–24^. For example, the following matrix can represent each unit cell:

1 $${M}_{i}=\left[\begin{array}{cc}A& B\\ C& D\end{array}\right]\left[\begin{array}{cc}1& 0\\ {y}_{D}& 1\end{array}\right]\left[\begin{array}{cc}A& B\\ C& D\end{array}\right],$$

where $A=\cos\left(k\frac{{d}_{i}}{2}\right), B=j {Z}_{i} \sin\left(k\frac{{d}_{i}}{2}\right), C=\frac{j}{{Z}_{i}}\sin\left(k\frac{{d}_{i}}{2}\right),$ $D=A, k=\omega /c$ is an acronym for the wave number. $\rho$ points to the density. $c$ is the speed of sound. ${Z}_{i}$ refers to the impedance of the acoustic waves:

2 $${Z}_{i}=\frac{\rho c}{{{\text{S}}}_{{\text{i}}}}.$$

In this closed resonator, the propagation of the acoustic wave at its end is zero. So, the admittance ($${y}_{D})$$ of the structure of the incident wave is^22^:

3 $${y}_{D}=j \frac{j}{{Z}_{D}} tan\left(k{d}_{D}\right).$$

The following Bloch’s equation describes the relation of the acoustic wave dispersion of a unit cell of the proposed periodic structure^25^:

4 $${\text{cos}}\left(Kd\right)={\text{cos}}\left(k{d}_{1}\right)- \frac{M}{2}{\text{sin}}\left(k{d}_{1}\right){\text{tan}}\left(k{d}_{2}\right),$$

where $K$ and k are the Bloch and wave vectors, respectively, $d= {d}_{1}+{d}_{2}$, $M=\frac{{S}_{2}}{{S}_{1}}$. The transmittance (T) is as follows:

5 $$t=\frac{2{\mathrm{\varnothing }}_{1}}{\left({A}_{11}+{A}_{12}{\mathrm{\varnothing }}_{1}\right){\mathrm{\varnothing }}_{1}+\left({A}_{21}+{A}_{22}{\mathrm{\varnothing }}_{1}\right)},$$

6 $$T\left(\mathrm{\%}\right)=100*{\left|t\right|}^{2}.$$

### Using Green method

The Green method will be used to check if the results of UATMM are correct. At the end of each closed branch resonator, acoustic velocity (u) is zero. The closed branch resonator is grafted along the horizontal tube periodically. The set of interface spaces of all connections of the finite guides is reduced to M = (0) as a single interface^26^. The response function of an inverse interface of a unit cell is^26,27^:

7 $${g}^{-1}\left(\mathrm{0,0}\right)=-2{F}_{1}+{g}_{R}^{-1}\left(\mathrm{0,0}\right),$$

where ${g}_{R}^{-1}\left(\mathrm{0,0}\right)$ is the Green’s surface function of the closed-branched tube according to the conditions of the boundary. The dispersion relation of the infinite periodic waveguide can be written as:

8 $${\text{cos}}\left(\mathit{Kd}\right)={\text{cos}}\left(k{d}_{1}\right)- \frac{1}{2}\frac{{z}_{1}}{\upomega }{\text{sin}}\left(k{d}_{1}\right){g}_{R}^{-1}\left(\mathrm{0,0}\right),$$

For closed resonators, ${g}_{R}^{-1}\left(\mathrm{0,0}\right)$ can be written as:

9 $${g}_{R}^{-1}\left(\mathrm{0,0}\right)=- \frac{{F}_{1}{S}_{2}}{{C}_{2}}=\upomega {y}_{2}{\text{tan}}\left(k{d}_{2}\right),$$

From Eqs. (8) and (9), the dispersion relation can be written as:

10 $${\text{cos}}\left(Kd\right)={\text{cos}}\left(k{d}_{1}\right)- \frac{M}{2}{\text{sin}}\left(k{d}_{1}\right){\text{tan}}\left(k{d}_{2}\right),$$

which is precisely the exact dispersion relation obtained by UATMM in Eq. (4).

### Ethics declarations

This article does not contain any studies involving animals or human participants performed by any authors.

## Results

**Figure 2:**
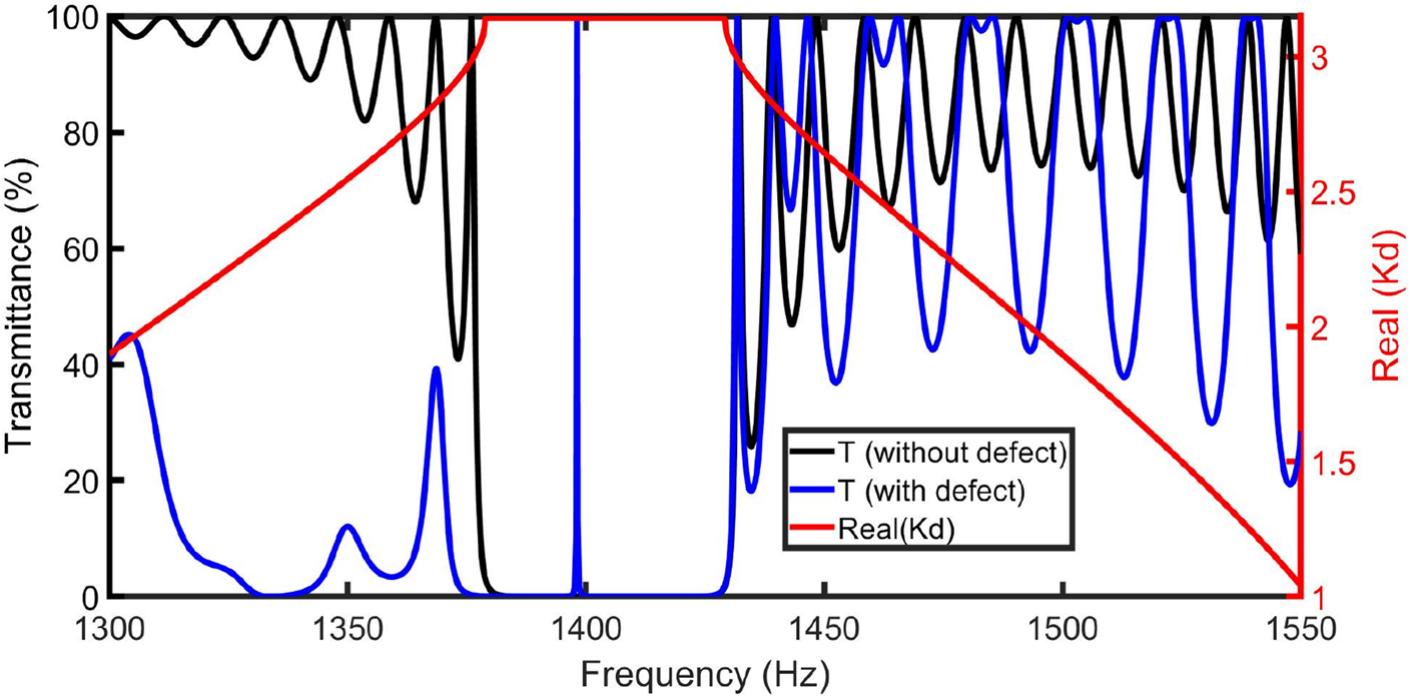
The band structure (red spectrum), the transmittance spectra of the closed resonator system without defect (black spectrum), and with an air cavity (blue spectrum) at *d*_*1*_ = 0.6 m, *S*_*D*_ = 0.73 m^2^, *d*_*2*_ = 0.15 m, *S*_*1*_ = 1 m^2^, *d*_*D*_ = 0.45 m, *S*_*2*_ = 0.75 m^2^, and *N* = 10.

The proposed sensor consists of (${M}^{N}{M}_{D}{M}^{N}$) with *N* = 10. The initial geometrical conditions are selected as *S*_*1*_ = 1 m^2^, *S*_*D*_ = 0.73 m^2^, *d*_*2*_ = 0.15 m, *d*_*1*_ = 0.6 m, *d*_*D*_ = 0.45 m, and *S*_*2*_ = 0.75 m^2^. The transmittance of the acoustic wave through the proposed novel gas sensor using periodically closed resonators has been studied as clearly in Fig. 2. By plotting the transmittance spectrum without the defect resonator, a Pn-BG extended from 1378.9 to 1429.1 Hz, with an intensity of 0%. The transmittance before and after the bandgap (width and intensity of ripples) depends on the constructive and destructive interferences of reflected waves at each interface due to the multiple Bragg scattering. The Bloch vector (*K*) within this range of frequencies is complex. So, the waves are evanescent. Real(*K*) is used to investigate changes in the phase of the pass band propagated wave. The black and red spectra in Fig. 2 clearly show that Bloch wavenumber dispersion and the Pn-BG coincide.

Inserting a defect-closed resonator at the center of the design causes the excitation of a sharp resonant peak with a minimal bandwidth inside the Pn-BG at 1398.16 Hz using an air sample.

**Figure 3:**
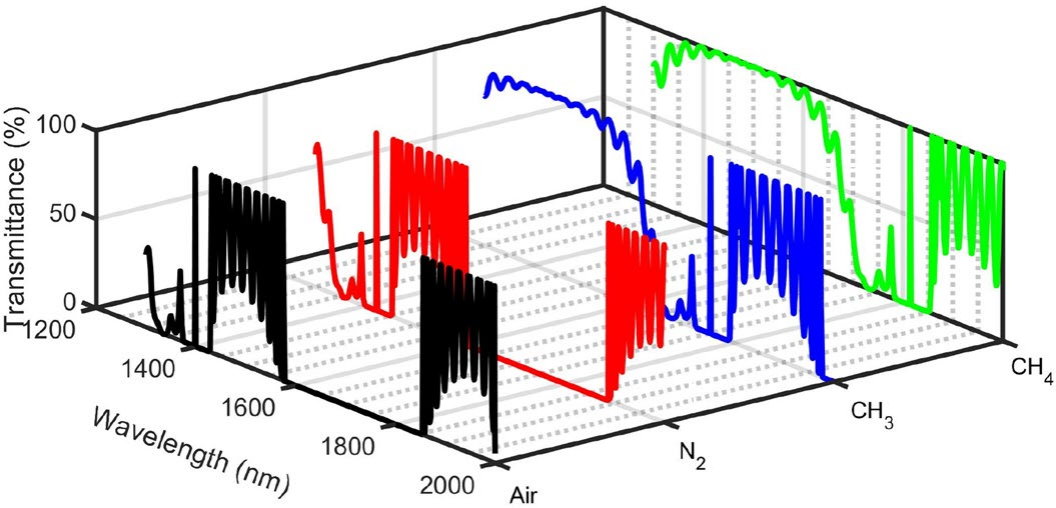
The transmittance spectra of the closed resonator system using different gas samples at *d*_*1*_ = 0.6 m, *N* = 10, *d*_*2*_ = 0.15 m, *S*_*1*_ = 1 m^2^, *d*_*D*_ = 0.45 m, *S*_*D*_ = 0.73 m^2^, and *S*_*2*_ = 0.75 m^2^.

In Fig. 3, the excited peaks and the Pn-BG shift toward higher frequencies as the speed of sound in the sample increases and its density decreases (Table 1). This behavior is known as the blueshift of the peak. To see the peak dependence on the type of gas sample, we change the sample from air to N_2_, NH_3_, and CH_4_. By replacing the air sample with N_2_, NH_3_, and CH_4_, the position of the excited peak is changed from 1398.16 to 1422.61 Hz, 1752.8 Hz, and 1813.94 Hz, respectively. The intensities of the excited peaks are very high (99.9%) due to the high localization of acoustic waves inside the defect-closed resonator. The dependence of the excited peaks on the acoustic velocity can be explained according to the standing wave equation:

**Table 1:** Acoustic properties of gas samples^16^.

Gas sample	Density ($\rho$) (kg/m^3^)	Acoustic speed ($c$) (m/s)
Air	1.2047	343
N_2_	1.165	349
NH_3_	0.7069	430
CH_4_	0.659	445

11 $$2d=\frac{n c}{{f}_{R}},$$

where *d* is the length of the defect-closed resonator*, n* is an integer*, C* is acoustic velocity*,* and ${f}_{R}$ is the frequency of the excited peak.

Any detector’s sensitivity (*S*) is calculated by the rate of change of the peak’s frequency ($\Delta {f}_{R}$) and acoustic speed (Hz m^−1^ s) as the following equation^28^:

12 $$S =\frac{\Delta {f}_{R}}{\Delta C}=\left|\frac{{f}_{CH4}-{f}_{air}}{{C}_{CH4}-{C}_{air}}\right|.$$

Besides, the *FoM* is expressed as^29^:

13 $$FoM=\frac{S}{FWHM},$$

where *FWHM* is the bandwidth of the defect peak. Also, the *Q-factor* and *LoD* can be calculated as follows^28,29^:

14 $$\begin{array}{l}Q-factor=\frac{{f}_{R}}{FWHM}\end{array},$$

15 $$LoD=\frac{{f}_{R}}{20 S Q}.$$

**Figure 4:**
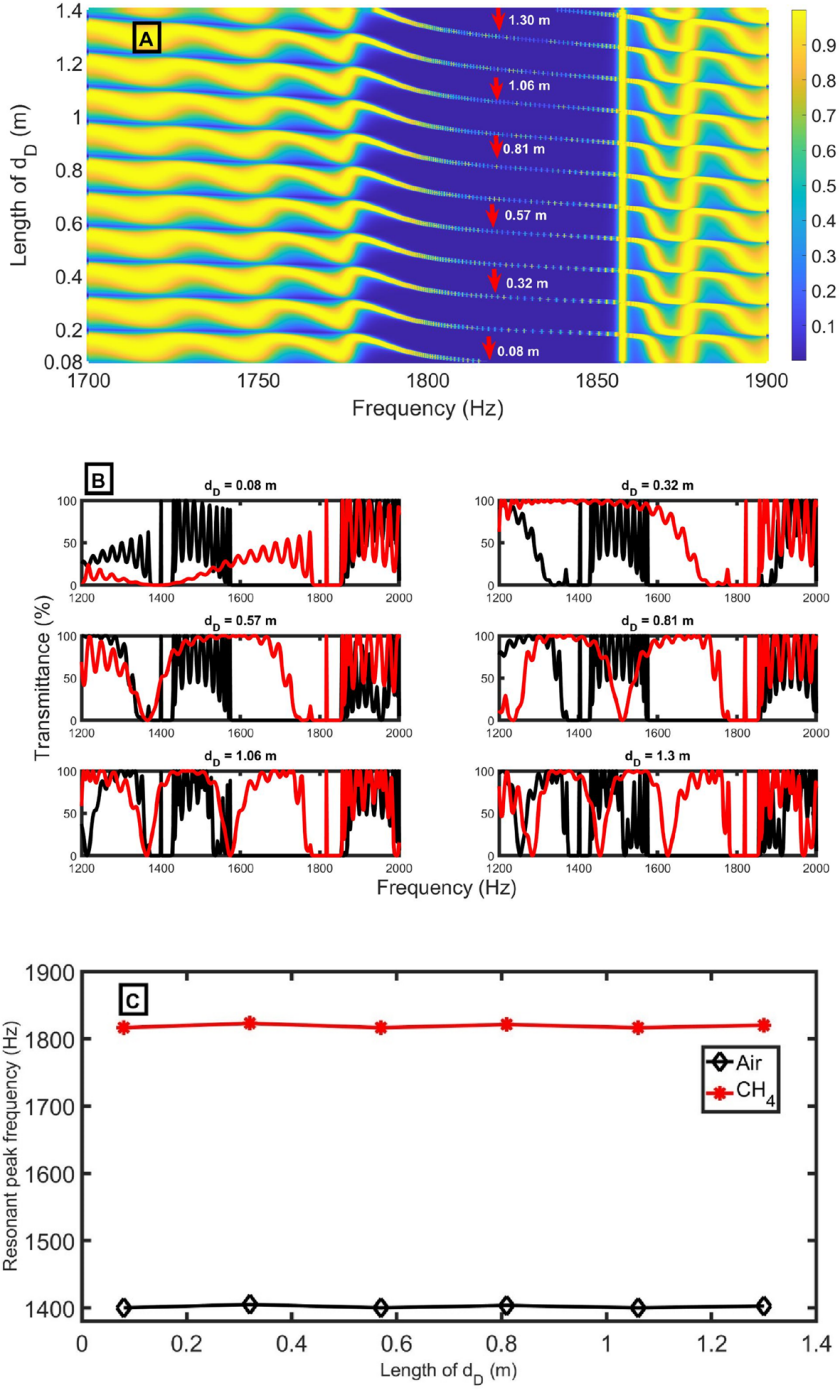
(**A**) transmittance intensity versus frequency as a function of the length of *d*_*D*_ using CH_4_ sample, (**B**) transmittance intensity versus frequency at selected values of *d*_*D*_ for air (black lines) and CH_4_ (red lines) samples, and (**C**) resonant peak positions for air (black lines) and CH_4_ (red lines) samples at different lengths of *d*_*D*_ at d_1_= 0.6 m, *N* = 10, d_2_= 0.15 m, S_2_= 0.75 m^2^, *S*_*1*_ = 1 m^2^, and *S*_*D*_ = 0.73 m^2^.

As expected, increasing the length of the defect-closed resonator ${d}_{D}$ does not reflect the position of Pn-BG, as evident in Fig. 4A. This expectation was built on the fact that the Pn-BG depends only on the potential (acoustical and geometrical) contrast between the layers in each unit cell, not the defect cell. Increasing the ${d}_{D}$ only reflects on the shape of the Pn-BG edges, as apparent in Fig. 4B. In Fig. 4B, we selected some values of ${d}_{D}$ that makes ${f}_{R}$ in the middle of the Pn-BG because the central resonance is highly responsive to slight changes in the sample and has the lowest FWHM. On the other hand, increasing the ${d}_{D}$ significantly impact the position (Eq. 11) and shape of the resonant peak. Increasing the length of the defect-closed resonator shifts the resonant peak to lower frequencies until it goes out from Pn-BG, another peak comes from the right, and so on. Besides, it was observed that the peak shift ($\Delta {f}_{R}$) seems constant. This independence of peak shift on the length of the defect-closed resonator may be considered an advantage because it gives flexibility in selecting a suitable length with the same peak shift (same sensitivity), as explicit in Fig. 4C. The resonant peak frequency for air and CH_4_ samples slightly changes, and $\Delta {f}_{R}$ seems to be constant with increasing* d*_*D*_*.* The resonant peak shift is expected to increase *d*_*D*_ due to the increasing interaction between acoustic waves and sample molecules, as in multilayer PnCs^29,30^. This difference between the effect of increasing the defect length inside multilayers and a lateral defect resonator is that increasing the defect length inside multilayers increases the path that waves will travel. As a result, the interaction between the incident wave and the sample inside the defect increases until a saturation occurs. So, the resonant peak shift increases with the defect length inside multilayers. But in our case, the defect resonator is lateral, and any increase in its length isn’t in the path of the incident acoustic waves. Besides, the impedance of the defect closed resonator doesn’t depend on the length (Eq. 2). For these reasons, the resonant peak shift seems to be constant.

**Figure 5:**
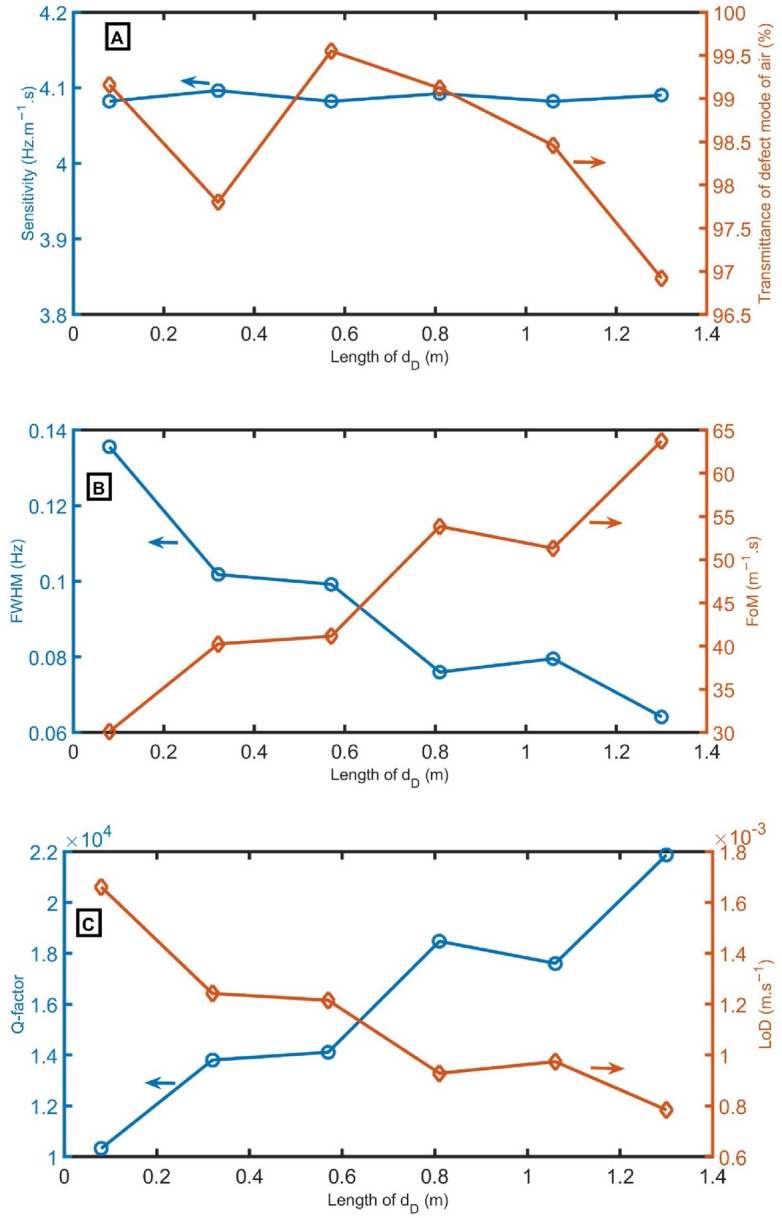
(**A**) resonant peak position for air (black lines) and CH_4_ (red lines) samples, (**B**) sensitivity and transmittance intensity, and (**C**) *FWHM* of defect mode for air sample and *FoM* as a function of the length of *d*_*D*_ at *N* = 10, *d*_*2*_ = 0.15 m, *d*_*1*_ = 0.6 m, *S*_*2*_ = 0.75 m^2^, *S*_*1*_ = 1 m^2^, and *S*_*D*_ = 0.73 m^2^.

In Fig. 5 A–C, sensitivity, transmittance intensity, *FWHM* of defect mode for air sample, *FoM*, *Q-factor*, and *LoD* as a function of the length of *d*_*D*_ are calculated. In Fig. 5A, the sensitivity and transmittance intensity are obtained as a function of the length of *d*_*D*_. As the sensitivity is directly proportional to the resonant peak shift (Eq. 12), with changing the *d*_*D*_ from 0.08 to 0.32 m, 0.57 m, 0.81 m, 1.06 m, and 1.3 m, the sensitivity slightly changes. The air sample is used to investigate the intensity of peaks as an indicator. The peak’s transmittance ranges from 96.9 to 99.6% for all studied lengths. Figure 5B shows the *FWHM* and *FoM* as a function of the original lengths of *d*_*D*_. The lowest *FWHM* (0.064 Hz) and highest *FoM* (63.7 m^−1^ s) are recorded at a length of 1.3 m. As apparent in Fig. 5C, the highest *Q-factor* (21,867) and lowest *LoD* (8 × 10^–4^ m s^−1^) are recorded at a length of 1.3 m. So, the length of *d*_*D*_ = 1.3 m will be used in the following studies.

**Figure 6:**
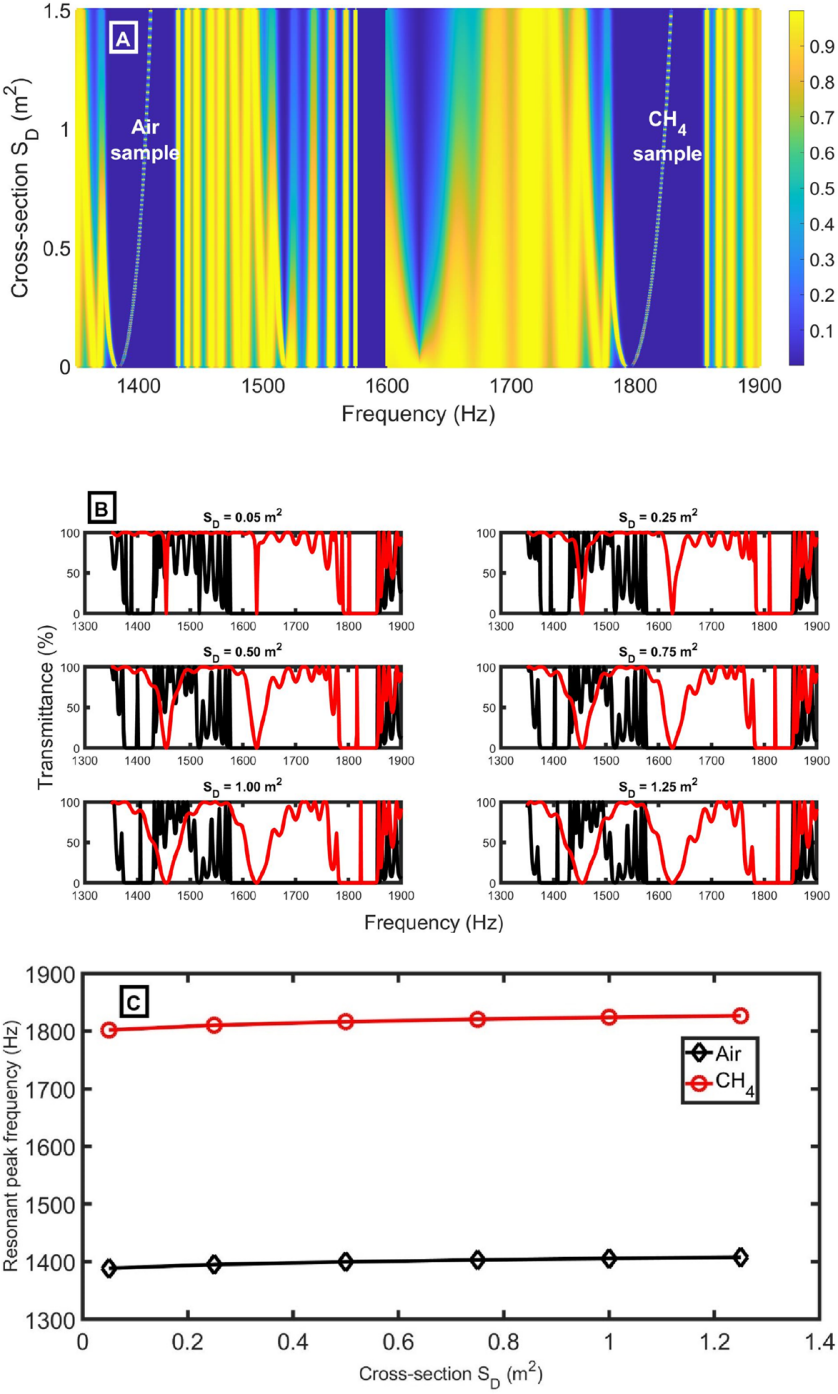
(**A**) transmittance intensity versus frequency as a function of the cross-section *S*_*D*_ using CH_4_ sample, (**B**) transmittance intensity versus frequency at selected values of *S*_*D*_ for air (black lines) and CH_4_ (red lines) samples, and (**C**) resonant peak positions for air (black lines) and CH_4_ (red lines) samples at different cross-section *S*_*D*_ at *N* = 10, *d*_*2*_ = 0.15 m, *d*_*1*_ = 0.6 m, *S*_*2*_ = 0.75 m^2^, *S*_*1*_ = 1 m^2^, and *d*_*D*_= 1.3 m.

Similar to the effect of length ${d}_{D}$ on the PnBG, increasing the cross-section of the defect-closed resonator ${S}_{D}$ does not reflect on the position of Pn-BG, as clear in Fig. 6A. Changing the ${S}_{D}$ only reflects on the shape of the Pn-BG edges, as clear in Fig. 6B. However, increasing the ${S}_{D}$ significantly impact the position of the resonant peak. By increasing the ${S}_{D}$, the resonant peak is shifted to higher frequencies. As clear in Fig. 6C, by increasing the ${S}_{D}$ from 0.05 to 1.25 m^2^, the $\Delta {f}_{R}$ slightly increases from 412.98 to 418.66 Hz*.* As the impedance is inversely proportional to the cross-sectional area (Eq. 2), increasing S_D_ decreases the impedance of the defect-closed resonator. So, the interaction between the acoustic wave and the sample inside the defect-closed resonator increases. So, the resonant peak position for air and CH_4_ samples slightly increases with increasing S_D_, and the $\Delta {f}_{R}$ slightly increases.

**Figure 7:**
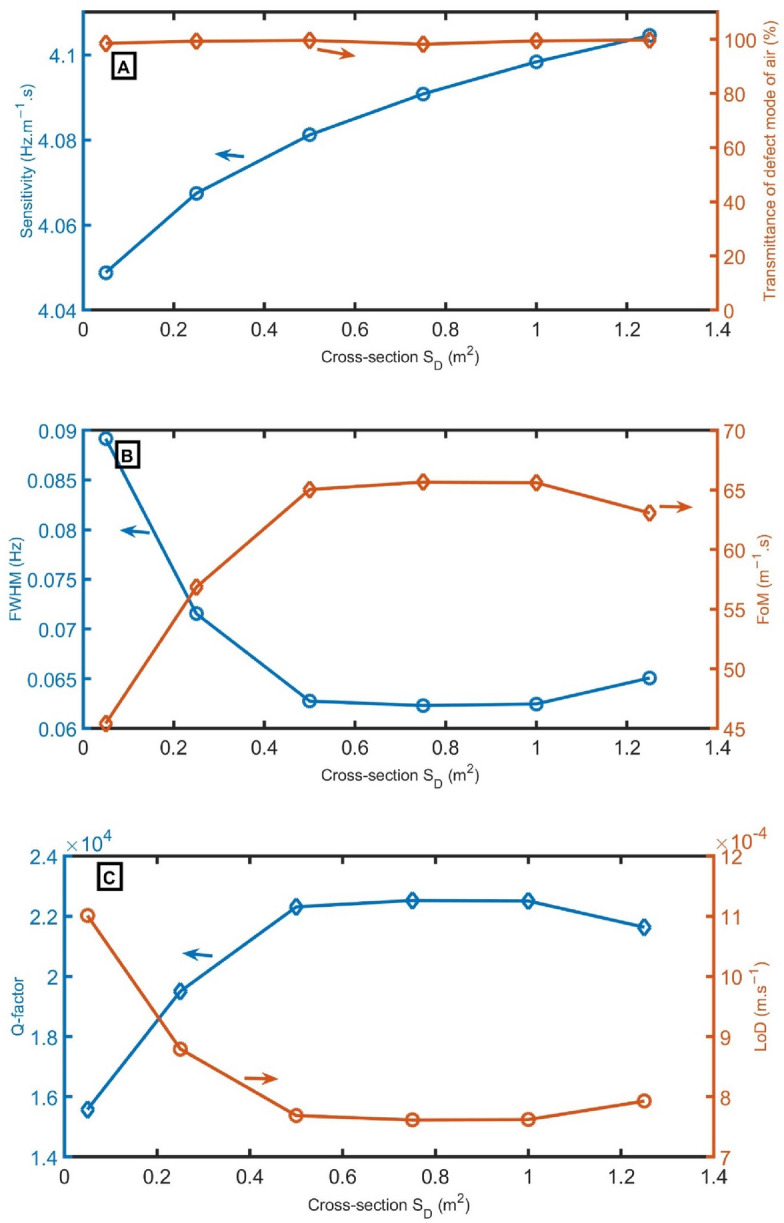
(**A**) sensitivity and transmittance intensity, (**B**) *FWHM* of defect mode for air sample and *FoM*, and (**C**) *Q-factor* and *LoD* as a function of the cross-section of *S*_*D*_ at *N* = 10, *d*_*2*_ = 0.15 m, *d*_*1*_ = 0.6 m, *S*_*1*_ = 1 m^2^, *d*_*D*_ = 1.3 m, and *S*_*2*_ = 0.75 m^2^.

Next, to evaluate the effect of cross-section *S*_*D*_, the sensor’s performance is studied by changing *S*_*D*_ from 0.05 m^2^ to 0.25 m^2^, 0.50 m^2^, 0.75 m^2^, 1.00 m^2^, and 1.25 m^2^, leaving other geometrical conditions unchanged. With increasing *S*_*D*_ from 0.05 m^2^ to 1.25 m^2^, a slight increase in the sensitivity from 4.05 to 4.11 Hz m^−1^ s can be observed in Fig. 7A. The peak’s transmittance ranges from 98.2 to 99.7% with increasing *S*_*D*_. In Fig. 7B, the lowest *FWHM* (0.062 Hz) and highest *FoM* (65.7 m^−1^ s) are recorded at a cross-section *S*_*D*_ of 0.75 m^2^. As explicit in Fig. 7C, the highest *Q-factor* (22,522) and lowest *LoD* (7.6 × 10^–4^ m s^−1^) are recorded at a cross-section *S*_*D*_ of 0.75 m^2^. The only reason why this enhancement at the cross-section *S*_*D*_ of 0.75 m^2^ is because at this *S*_*D*_ of 0.75 m^2^, the resonance is very close to the middle of Pn-BG, and the FWHM is very small at the center of the Pn-BG relative to at the edges. As a result, the *S*_*D*_ of 0.75 m^2^ is optimum.

**Figure 8:**
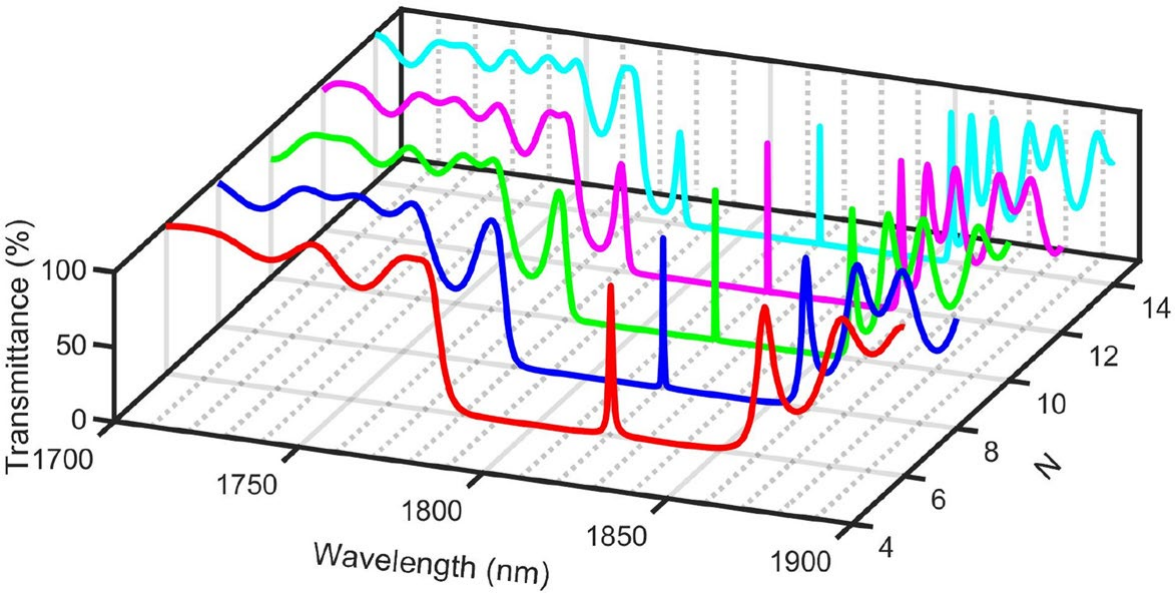
The transmittance spectra using different *N* = 10 at *d*_*2*_ = 0.15 m, *d*_*1*_ = 0.6 m, *d*_*D*_ = 1.3 m, *S*_*D*_ = 0.75 m^2^, *S*_*1*_ = 1 m^2^, and *S*_*2*_ = 0.75 m^2^.

Figure 8 shows that the Pn-BG and resonant peak frequencies for the CH_4_ sample don’t change with increasing *N* from 6 to 14 periods*,* and the resonant peak shift remains constant. Increasing *N* can be considered a double-edged sword. Increasing *N* from 6 to 10 periods enhances the Bragg-scattering. As a result, the edges of the Pn-BG become sharper, and the bandwidth of the resonant peak decreases. On the other hand, in periods higher than 10, the reflectance increases, and the transmittance decreases.

**Figure 9:**
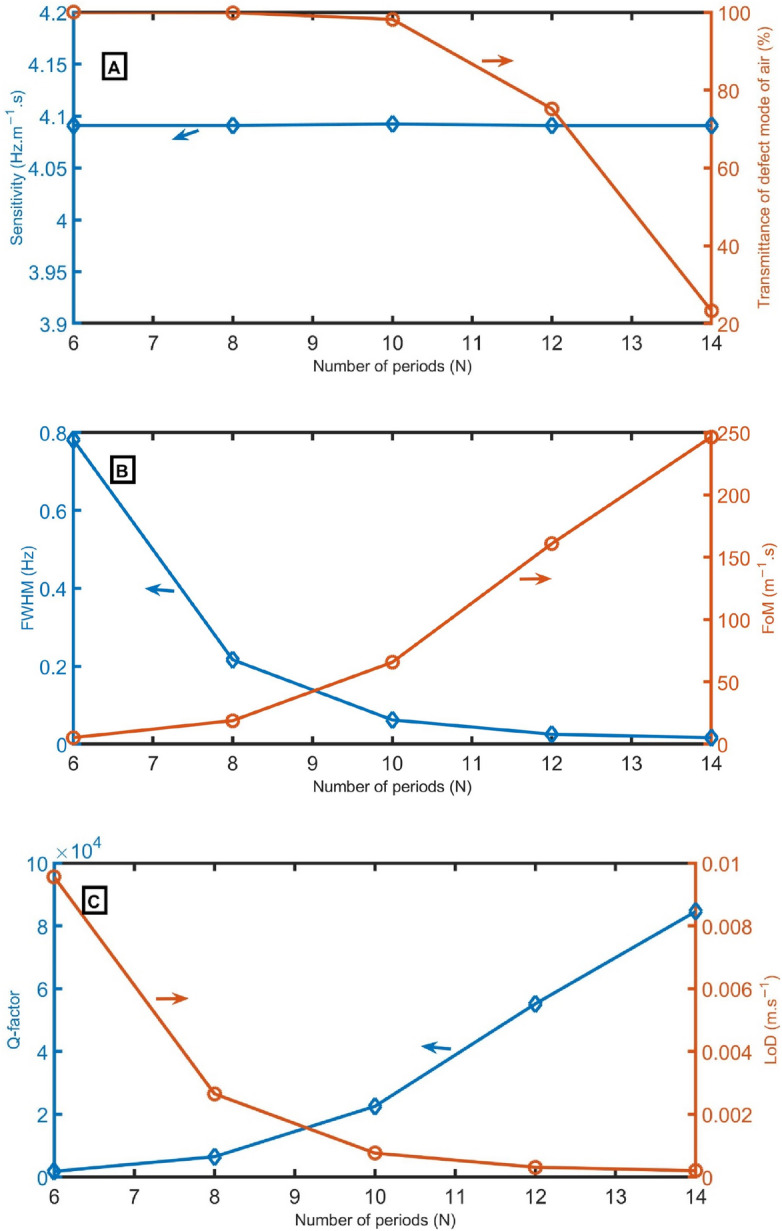
(**A**) sensitivity and transmittance intensity, (**B**) *FWHM* of defect mode for air sample and *FoM*, and (**C**) *Q-factor* and *LoD* as a function of N at *S*_*D*_ = 0.75 m^2^, *d*_*2*_ = 0.15 m, *d*_*1*_ = 0.6 m, *S*_*1*_ = 1 m^2^, *d*_*D*_ = 1.3 m, and *S*_*2*_ = 0.75 m^2^.

In Fig. 9 A–C, sensitivity, transmittance intensity, *FWHM* of defect mode for air sample, *FoM*, *Q-factor*, and *LoD* are calculated as a function of the number of periods (N). From Fig. 9A, the sensitivity and transmittance intensity are obtained as a function of the number of periods. With changing the number of periods from 6 to 8 periods, 10 periods, 12 periods, and 14 periods, the sensitivity records the same value (4.09 Hz m^−1^ s). When the number of periods increases from 6 to 10 periods, the peak’s transmittance slightly decreases from 100 to 98%. By increasing the number of periods above 10 periods, the peak’s transmittance strongly decreases. Figure 9B shows the *FWHM* and *FoM* versus the number of periods. *FWHM* strongly decreases with increasing periods from 6 to 10 periods, and FoM slightly increases with increasing periods from 6 to 10 periods due to their inverse relationship (Eq. 13). Then, *FWHM* slightly decreases, but *FoM* strongly increases. As clear in Fig. 9C, the *Q-factor* slightly increases with increasing N from 6 to 10 periods, and *LoD* strongly decreases with increasing periods from 6 to 10. Then, the *Q-factor* strongly increases, and *LoD* slightly decreases. *N* of 12 periods will be used in the following studies to ensure high *FoM* and *Q-factor* with acceptable transmittance.

**Figure 10:**
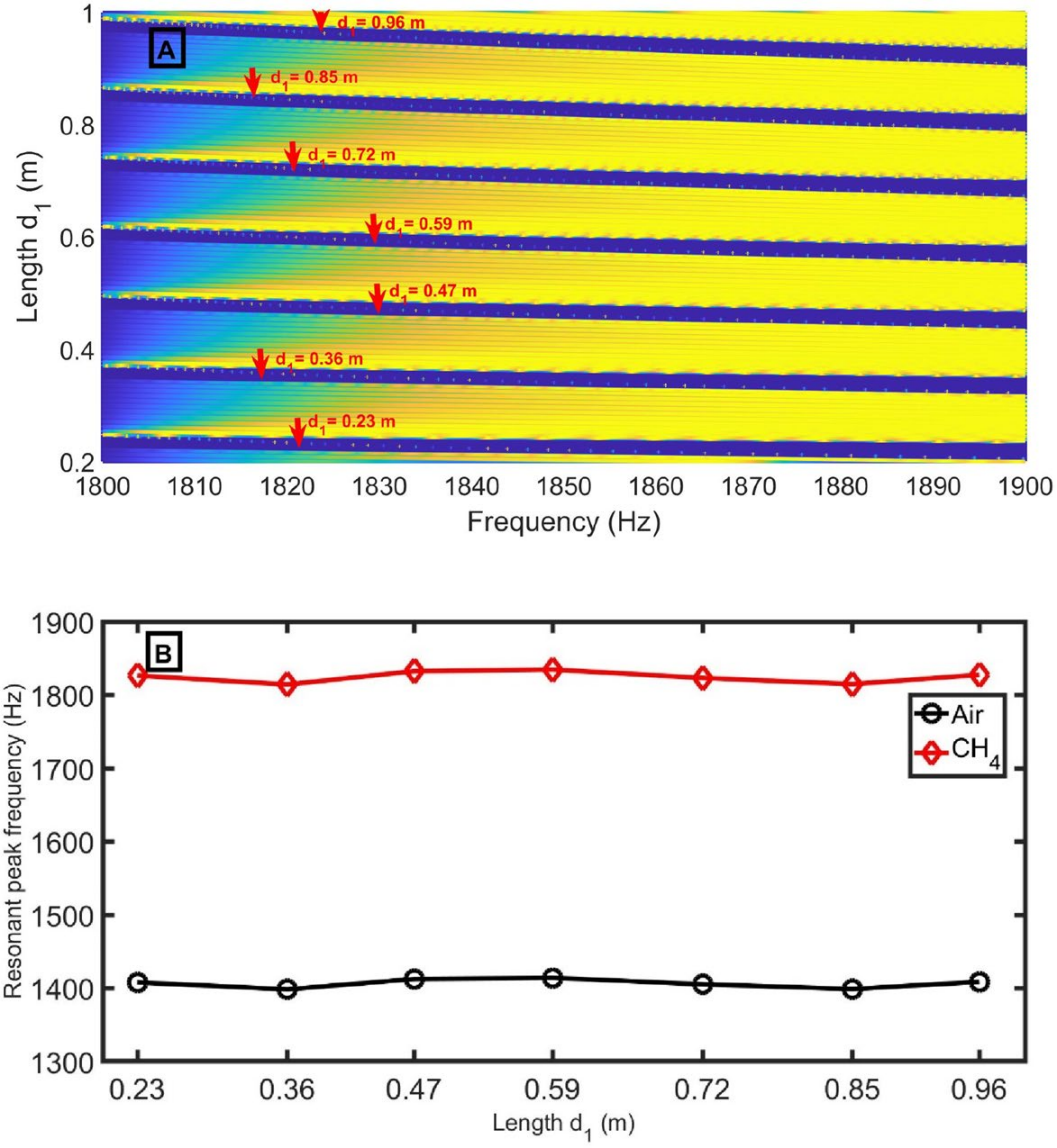
(**A**) transmittance intensity versus frequency as a function of the length *d*_*1*_ using CH_4_ sample, and (**B**) resonant peak positions for air (black lines) and CH_4_ (red lines) samples at different length *d*_*1*_ at *N* = 12, *S*_*D*_ = 0.75 m^2^, *d*_*2*_ = 0.15 m, *S*_*2*_ = 0.75 m^2^, *S*_*1*_ = 1 m^2^, and *d*_*D*_ = 1.3 m.

**Figure 11:**
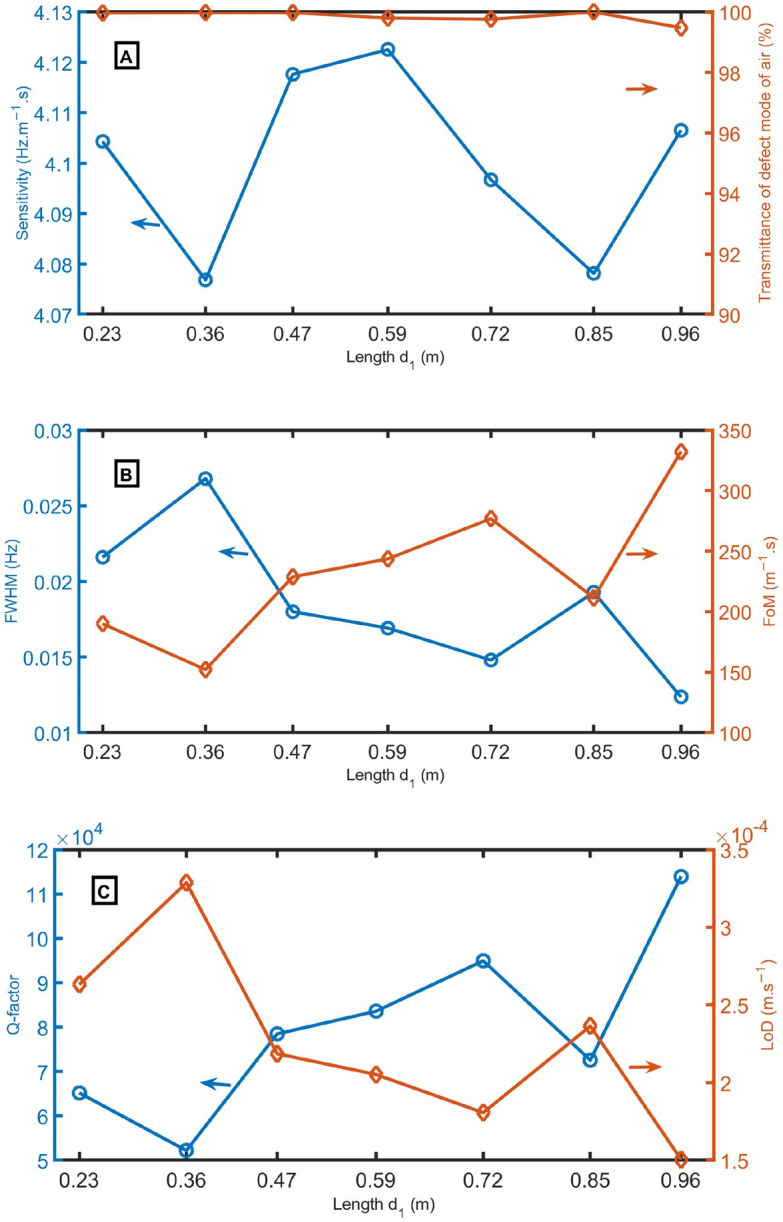
(**A**) sensitivity and transmittance intensity, (**B**) *FWHM* of defect mode for air sample and *FoM*, and (**C**) *Q-factor* and *LoD* as a function of length d_1_ at N = 12 periods, *d*_*1*_ = 0.6 m, *d*_*D*_ = 1.3 m, *d*_*2*_ = 0.15 m, *S*_*1*_ = 1 m^2^, *S*_*D*_ = 0.75 m^2^, and *S*_*2*_ = 0.75 m^2^.

Figure 10A clears the transmittance intensity versus frequency as a function of the length *d*_*1*_ using the CH_4_ sample. In the present study, the length of *d*_*1*_ changes only from 0.23 to 0.96 m because increasing the unit cell length may be a disadvantage of the proposed model. The Pn-BG is shifted to lower frequencies with increasing the length *d*_*1*_. The resonant peak shift slightly changed and recorded the highest shift at the length of 0.59 m, according to Fig. 10B. As shown in Fig. 11A, the sensitivity slightly increases with the length of *d*_*1*_. Besides, the transmittance ranges from 99.7 to 100%. The *FWHM* in Fig. 11B strongly depends on the position of peaks inside Pn-BG and ranges from 0.0148 Hz to 0.0268 Hz. *FoM* and *Q-factor* are inversely proportional to the *FWHM*. According to Eqs. (13) and (14), both have behavior opposite to the *FWHM*. In Fig. 11C, the *LoD* has the same behavior as *FWHM* according to Eq. (15). The length of 0.96 m records the best FWHM, FoM, Q-factor, and LoD so that it will be the optimum.

**Figure 12:**
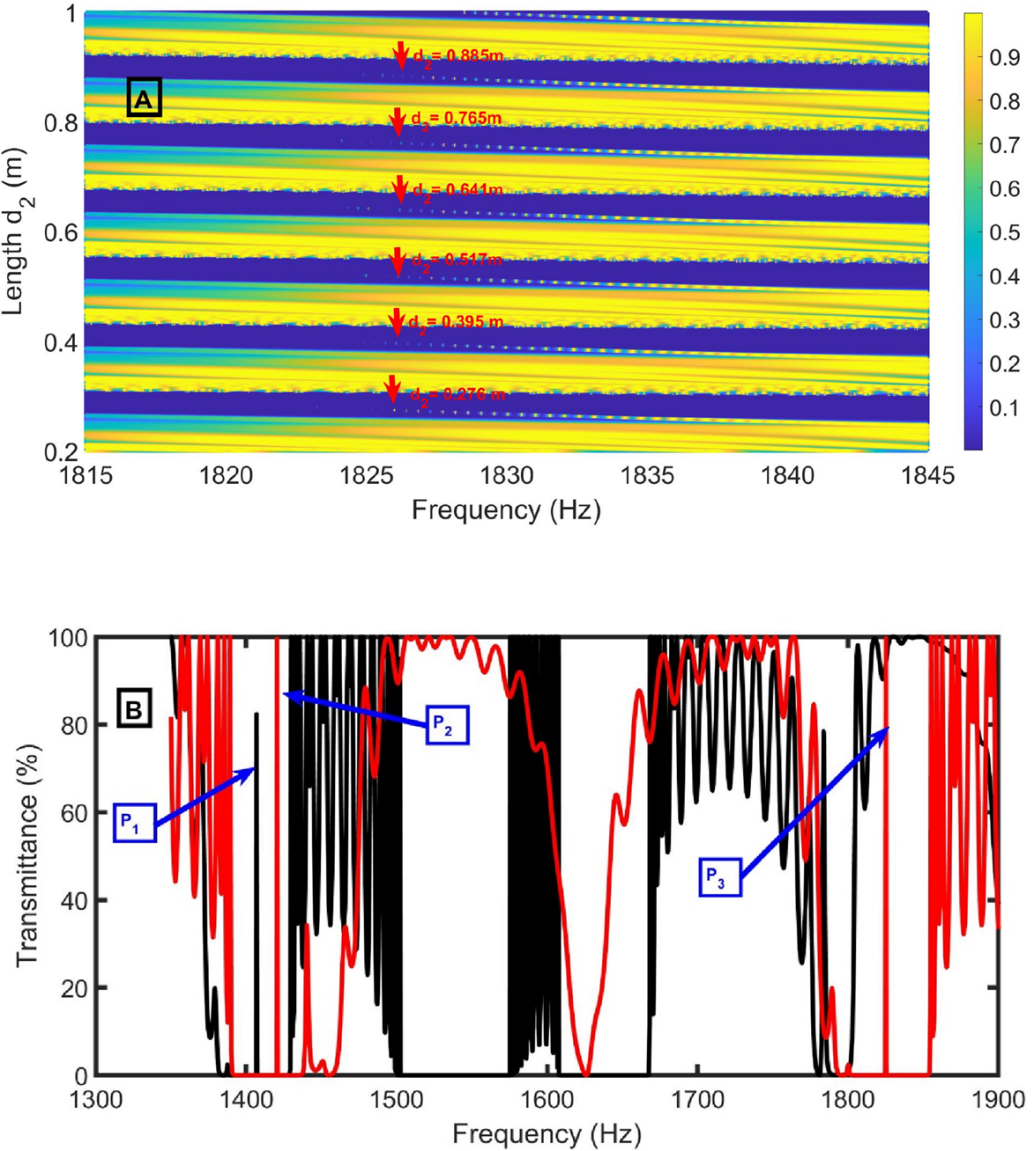
(**A**) transmittance intensity versus frequency as a function of the length *d*_*2*_ using CH_4_ sample, and (**B**) transmittance intensity versus frequency at selected values of *d*_*2*_ for air (black lines) and CH_4_ (red lines) samples at *N* = 12, *S*_*D*_ = 0.75 m^2^, *d*_*1*_ = 0.96 m, *S*_*2*_ = 0.75 m^2^, *S*_*1*_ = 1 m^2^, and *d*_*D*_ = 1.3 m.

The length of* d*_*2*_ varies from 0.2 to 1.0 m to study its effect on the transmittance spectra. By increasing the length of d_2_, the Pn-BG and peaks are red-shifted, as clear in Fig. 12A. The lengths of 0.276 m, 0.395 m, 0.517 m, 0.641 m, 0.765 m, and 0.885 m are selected to study the model’s performance at them. Unfortunately, when the transmittance spectra for air and CH_4_ are plotted at the length of 0.276 m, an undesired peak (P_2_) is found between the peaks of air and CH_4_ (P_1_ and P_3_), as clear in Fig. 12B. The same effects were observed at other lengths. So, a length of 0.15 m will be optimum.

**Figure 13:**
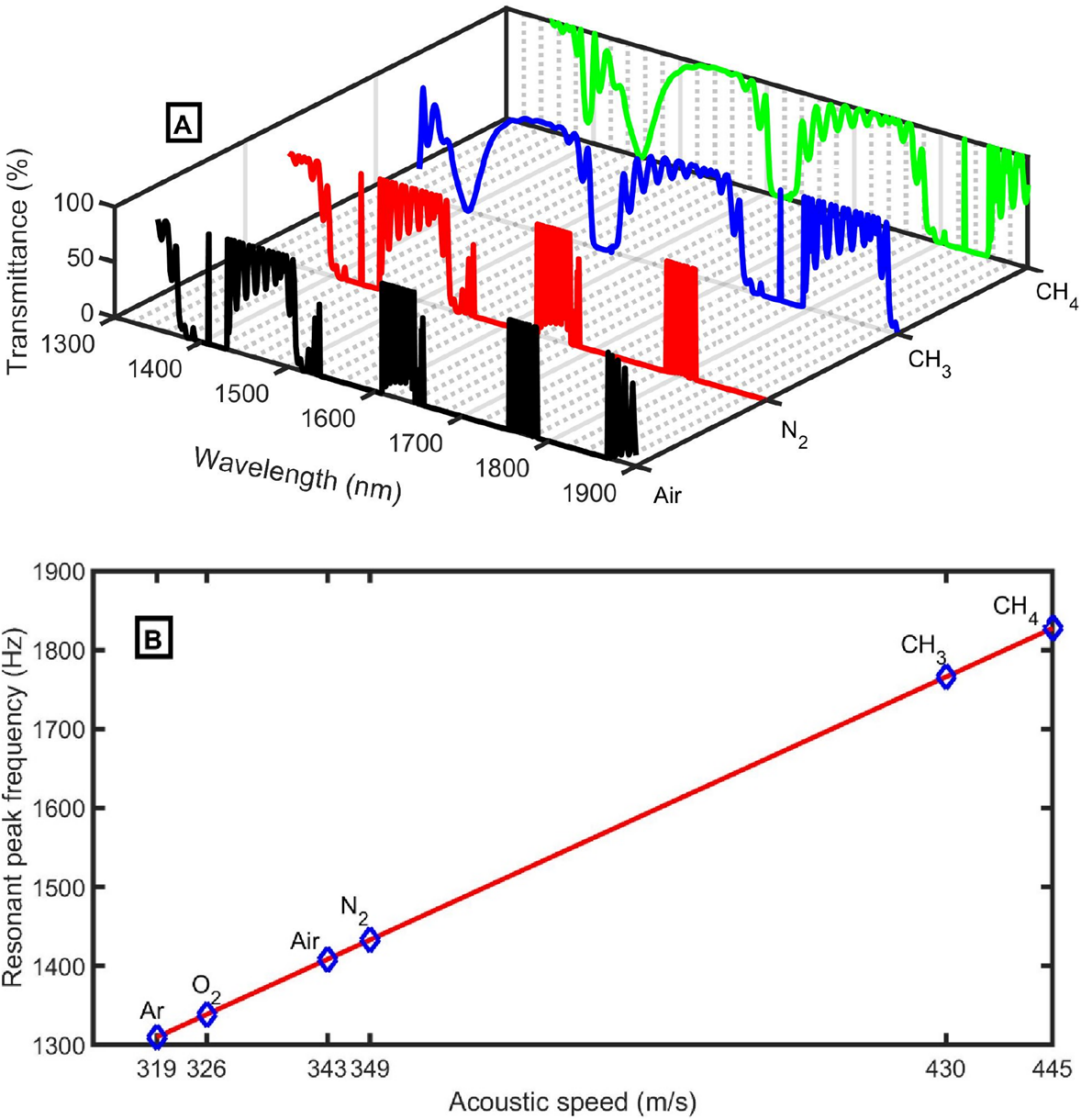
(**A**) The transmittance spectra of the closed resonator system, and (**B**) the resonant peak position versus acoustic speeds using different gas samples at optimum conditions at *N*=12, *d*_*1*_=0.96 m, *S*_*1*_ =1 m^2^, *d*_*2*_=0.15 m, *S*_*2*_=0.75 m^2^, *S*_*D*_ =0.75 m^2^, and *d*_*D*_=1.3 m.

Then, the transmittance spectra of the closed resonator system are studied in Fig. 13A using the selected geometrical parameters. By changing the air sample with N_2_, NH_3_, and CH_4_, the position of the excited peak changed from 1408.57 Hz, 1433.21 Hz, 1765.84 Hz, and 1827.44 Hz, respectively. The intensities of the excited peaks are very high (99.9%) due to the high localization of acoustic waves inside the defect-closed resonator at the selected geometrical parameters. The resonant peak position versus acoustic speeds using different gas samples at optimum conditions is clear in Fig. 13B. Ar ($\rho = 1.661 kg/{m}^{3}$ and *C* = *319 m/s*) and O^2^ ($\rho = 1.314 kg/{m}^{3}$ and *C* = *326 m/s*) samples are added in Fig. 13B to ensure the linearity of the sensor for different samples.

The following relation (Eq. 16) describes the linearity of the sensor with an average sensitivity of 4.07 Hz m^−1^ s:

16 $${f}_{R} = 4.107 C - 0.02049.$$

In Table 2, compared with other designs, the proposed closed system has achieved outstanding performance with a high sensitivity of 4.1 Hz m^−1^ s, a high *FoM* of 332 m^−1^ s, a very outstanding *Q-factor* of 113,962, and a small *LoD* of 0.0002 m s^−1^. Even though many previous studies with complicated structures and materials achieved better outcomes, most of them couldn’t achieve linearity (linear peak shift or constant sensitivity) as in our model. For example, Zaki et al.^31^ proposed a defective 1D-Pn-BG based on a high-sensitivity fano resonance, but the linearity was missed. Aliqab et al.^32^ suggested a sensor to detect sulfuric acid concentration using 1D-PnC. Their model recorded a good sensitivity, but the linearity between the peak shift and the acoustic speed was missed. Zaky et al.^11^ studied the ability to use the periodic cross-section of phononic tubes as gas sensors. This structure of periodic cross-section of phononic tubes recorded limited sensitivity (*S*) of 2.5495 Hz s m^−1^, limited *Q-factor* of 4077, and limited *FoM* of 9.16 s m^−1^.

**Table 2:** Comparison study.

Ref	S (Hz s m^−1^)	Q	FoM (s m^−1^)	Structure
2022^11^	2.55	4077	9.16	Binary-asymmetric periodic tubes
2023^33^	1.58	6790	33.7	Ternary-symmetric periodic tubes
2023^34^	5.8	5000	140	Branched open resonator
This work	4.1	113,962	323	Closed resonators

## Conclusion

The acoustic wave is better localized in the closed resonator by designing a phononic crystal with periodically closed resonators as a greenhouse gas sensor. This acoustic wave localization changes the peak position with any change in the acoustic properties of the analyte. Therefore, the proposed phononic crystal with periodically closed resonators as a greenhouse gas sensor is a good choice with many features as the following:

1. Compared with other designs, this sensor is more straightforward to fabricate than nano-dimensional structures. It is also cheaper than other sensors, making them more cost-effective for industrial applications.
2. This sensor can be a part of the factory chimney and continuously detect the concentration of hazardous gases’ path through it.
3. No recovery time is required.
4. High linearity.
5. The proposed closed resonator gas sensor records a high sensitivity of 4.1 Hz m^−1^ s, a high *FoM* of 332 m^−1^ s, a high *Q-factor* of 113,962, and a small *LoD* of 0.0002 m s^−1^.

## Data Availability

Requests for materials should be addressed to Zaky A. Zaky.
